# Trait and State Positive Emotional Experience in Schizophrenia: A Meta-Analysis

**DOI:** 10.1371/journal.pone.0040672

**Published:** 2012-07-18

**Authors:** Chao Yan, Yuan Cao, Yang Zhang, Li-Ling Song, Eric F. C. Cheung, Raymond C. K. Chan

**Affiliations:** 1 Neuropsychology and Applied Cognitive Neuroscience Laboratory, Key Laboratory of Mental Health, Institute of Psychology, Chinese Academy of Sciences, Beijing, China; 2 Graduate School, Chinese Academy of Sciences, Beijing, China; 3 Department of Applied Social Studies, City University of Hong Kong, Hong Kong Special Administrative Region, China; 4 College of Life Science and Bio-engineering, Beijing University of Technology, Beijing, China; 5 Castle Peak Hospital, Hong Kong Special Administrative Region, China; Baylor College of Medicine, United States of America

## Abstract

**Background:**

Prior meta-analyses indicated that people with schizophrenia show impairment in trait hedonic capacity but retain their state hedonic experience (valence) in laboratory-based assessments. Little is known about what is the extent of differences for state positive emotional experience (especially arousal) between people with schizophrenia and healthy controls. It is also not clear whether negative symptoms and gender effect contribute to the variance of positive affect.

**Methods and Findings:**

The current meta-analysis examined 21 studies assessing state arousal experience, 40 studies measuring state valence experience, and 47studies assessing trait hedonic capacity in schizophrenia. Patients with schizophrenia demonstrated significant impairment in trait hedonic capacity (Cohen’s *d* = 0.81). However, patients and controls did not statistically differ in state hedonic (valence) as well as exciting (arousal) experience to positive stimuli (Cohen’s d = −0.24 to 0.06). They also reported experiencing relatively robust state aversion and calmness to positive stimuli compared with controls (Cohen’s *d* = 0.75, 0.56, respectively). Negative symptoms and gender contributed to the variance of findings in positive affect, especially trait hedonic capacity in schizophrenia.

**Conclusions:**

Our findings suggest that schizophrenia patients have no deficit in state positive emotional experience but impairment in “noncurrent” hedonic capacity, which may be mediated by negative symptoms and gender effect.

## Introduction

Anhedonia, defined as the inability to experience pleasure, has been long considered a core deficit of negative symptoms in schizophrenia [1], [2]. In the past decade, more and more researchers have focused their attention on studying pleasure deficits in patients with schizophrenia. Different assessments were employed to examine trait and state positive emotional experience. Trait hedonic capacity is examined using interview-based measures and self-report trait measures while state positive emotional experience is assessed by using laboratory-based assessments [3].

In the past two decades, substantial evidence indicates that patients with schizophrenia have deficits in their trait hedonic capacity [3], [4], [5], [6]. However, few quantitative reviews have examined the extent to which these patients differ in trait hedonic capacity from healthy controls. There is only one quantitative review showing that schizophrenia was associated with low levels of extraversion [6]. However, the results were limited by the small number of articles and sample in the meta-analysis as well as the limited range of measures of trait anhedonia (only Eysenck personality inventory was included). In view of the growing number of self-reported scales (i.e. Chapman Physical/Social Anhedonia Scale [7], [8] etc) developed specifically to examine trait hedonic capacity in schizophrenia, it is necessary to revisit trait anhedonia in schizophrenia.

On the other hand, a lot of studies based on laboratory assessments surprisingly found that patients with schizophrenia showed comparative state positive emotional experience as healthy sample did [9], [10]. A most recent meta-analysis [9] on state emotional experience in patients with schizophrenia across laboratory studies showed that irrespective of the scale used, patients with schizophrenia and healthy controls did not statistically differ in their state subjective hedonic reaction to positive stimuli. This meta-analysis only examined valence rating in schizophrenia. However, arousal level, another important structure of emotional experience, was not fully examined [11], [12]. Kring and Moran suggested that valence-arousal affective circumplex exists in healthy controls as well as patients with schizophrenia and the pattern of arousal experience in schizophrenia is rather similar to that in healthy sample [10], [13]. However, there is little data supporting this claim. Findings from experimental studies examining emotional arousal in schizophrenia are relatively inconsistent [14], [15], [16], [17]. A number of studies did not find significant difference of state arousal experience between patients and healthy controls [14], [18], [19], [20]. Some other findings, however, indicated that patients with schizophrenia demonstrated impairment in their subjective arousal experience [16] and automatic arousal reaction (e.g. skin conductance) to emotional stimuli (especially neutral stimuli) [21], [22]. It is useful to clarify the nature of arousal experience in schizophrenia.

In view of the variation of positive emotional experience in schizophrenia, some confounding factors such as negative symptoms and gender should be taken into consideration. Empirical findings indicated that negative symptoms was closely link to positive emotional experience (especially trait hedonic capacity) [23], [24] and even the neurobiology of anhedonia in schizophrenia [25], [26]. For example, Suslow (2003) demonstrated that trait hedonic capacity was impaired in patients with anhedonic schizophrenia rather than those without anheonia [24]. Neuroimaging studies also suggested that the level of negative symptoms was associated with the reduction of activation in the striatum and prefrontal cortex in schizophrenia [27]. Considering the heterogeneity of the findings for positive affect in schizophrenia, understanding how negative symptoms mediate the effect of positive emotional experience would contribute to the understanding of the varied findings in positive affect in schizophrenia. Gender is another confounding factor to be considered. Compared with male patients, female patients have a later onset of illness, a more benign course and better medication response, better premorbid social functioning and less negative symptoms [10]. Women but not men with schizophrenia were found to exhibit comparable positive emotional experience, neural activity, and expressed emotion relative to healthy controls [28], [29], [30], [31]. Due to the limited knowledge of gender effect on positive affect in schizophrenia, elucidating how gender might influence positive emotional response in patients with schizophrenia might provide new insights in this field.

To our knowledge, no meta-analysis has been conducted to examine the extent of differences of arousal experience between patients with schizophrenia and healthy controls. The purpose of the current meta-analysis was to examine the extent of differences of both trait hedonic capacity and state positive emotional experience between schizophrenia patients and healthy controls. We primarily focused on positive affect with regards to both arousal and valence in schizophrenia.

## Methods

### Literature Search Strategies

Potential articles were identified through a comprehensive search using literature databases of EBSCOHost (PsychINFO, PsychACTICLE), MEDLINE, and Web of Knowledge between January 1980 and April 2012. The keywords^1^ used included: a word base of “schizophren*” or “bipolar disorder” and “anhedonia” or “anticipatory pleasure” or “consummatory pleasure” or “emotional experience” or “emotional expression” or “mood induc*” or “pleasure” or “positive emotional experience” or “positive emotional expression” or “pleasure scale” or “Chapman anhedonia scale” or “SHAPS” or “TEPS” or “GTS” or “FCPS” or “extraversion” or “positive affect” or “Eysenck personality” or ““pleasure” + “facial expression” or “heart rate” or “physiology” or “skin conductance”” (yielding 4634 articles). Another 23 articles were also obtained from the reference lists of the prior three reviews ([3], [9], [10]. In summary, these search procedures yielded an initial pool of 4,657 potential articles for inclusion.

The following exclusion criteria were used to select studies in the initial pool for the meta-analysis: 1) articles focusing on non-human subjects; 2) articles not published in peer-reviewed journals; 3) poorly-defined emotion induction paradigms and questionnaires; 4) articles not written or translated in English; 5) duplicated articles; 6) articles that did not report data on patients with schizophrenia or schizoaffective disorder and healthy controls; 7) articles with no available full text; 8) articles that did not focus on positive emotional experience.

**Figure 1:**
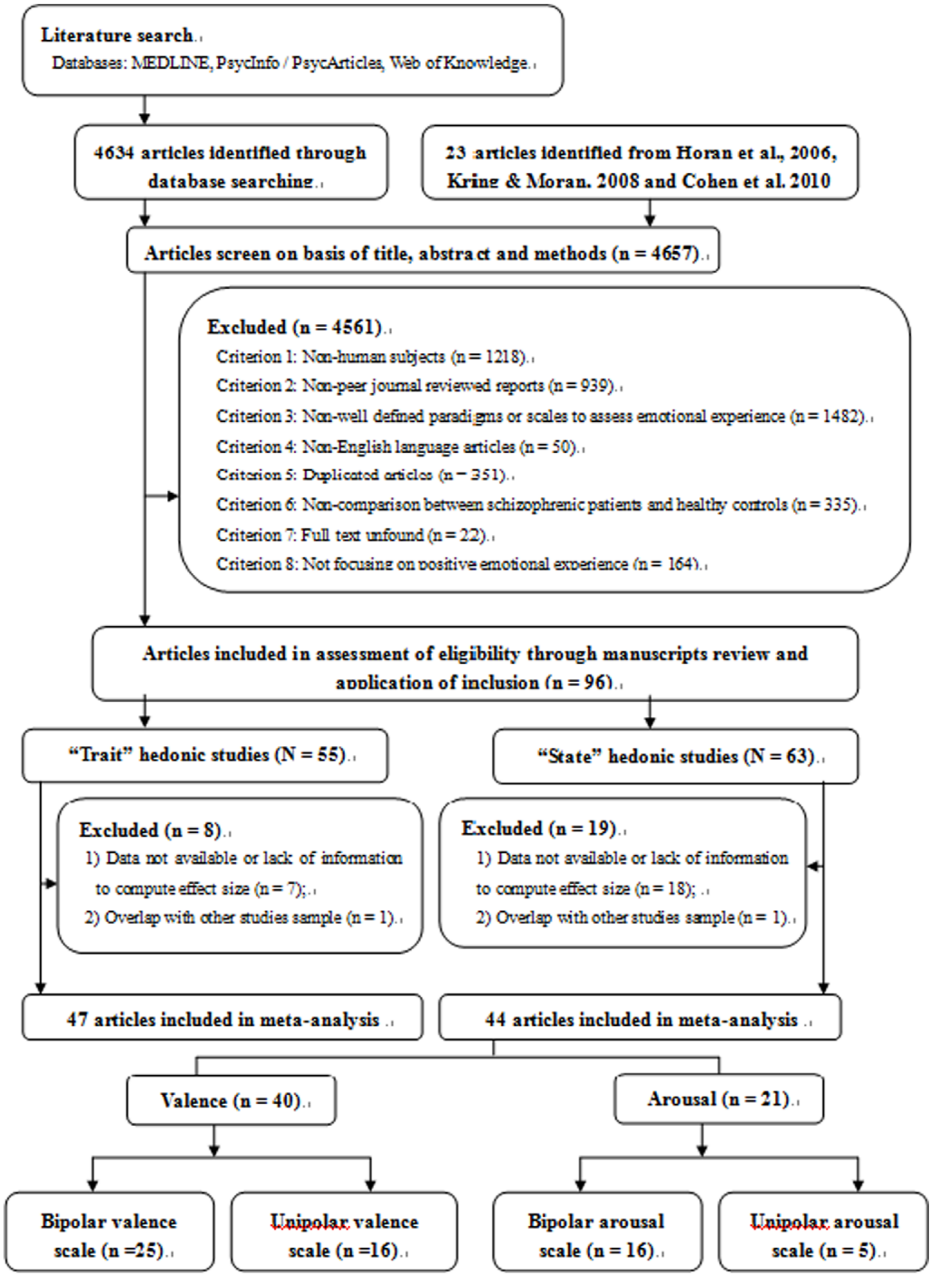
Flow diagram describing the process of articles searching and selection through exclusion and inclusion criteria.

This procedure yielded a study base of 96 published articles. Among these, 55 articles assessed trait hedonic capacity and 63 assessed state positive emotional experience in schizophrenia. For studies measuring trait hedonic capacity, data were not available or incomplete in seven articles to compute effect size and data in one study overlapped with another study [23], [32]. Since the healthy control data for Blandchard’s study [32] and Horan’s [23] study came from the same database, we only included data from Horan’s study. As a result, 47 studies were included in the final meta-analysis for trait hedonic capacity. For studies assessing state positive emotional experience, 18 articles did not have data or enough information to compute effect size and data in one study overlapped with another study [21], [33]. Similarly, we only included data from Kring and Neale [21].As a result, we had 44 articles to investigate state positive emotional experience using laboratory-based measures for the final meta-analysis (Valence (n = 40) vs. Arousal (n = 21)). (Details see in Figure 1).

### Data Encoding

The articles included in the meta-analysis were further divided into two categories: “trait” and “state” hedonic studies. “Trait” hedonic studies refer to studies employing trait self-reported measures to assess trait hedonic capacity. “State” hedonic studies refer to studies employing laboratory-based assessment to measure state positive emotional experience. Emotional experience can be divided into two independent structures: valence and arousal [11], [12]. Among these studies, 40 assessed the valence dimension of positive affect by asking participants to give hedonic rating and 21 measured arousal dimension of positive experience by asking participants to rate the extent of excitement. In the current study, we performed two sets of meta-analyses in terms of valence (overall hedonic rating) and arousal (overall exciting rating), respectively. Another consideration is the subjective rating scales used across the “state” hedonic studies. Based on the suggestion in Cohen et al’s, unipolar and bipolar scales were not combined in one meta-analysis because these two types of scale employed in “state” hedonic studies were based on fundamentally distinct theories of emotion (ie, “orthogonal” vs. “circumplex” models) [9], [34]. Unipolar scales refer to subjective rating scale assessing either state hedonic/exciting or aversive/calm emotion in separate scales, whereas bipolar scales refer to subjective rating scale set up with both extreme state hedonic/exciting and aversive/calm emotion on opposing ends of a continuum. Therefore, we further performed another four meta-analyses on studies using unipolar scales and bipolar scales (two for valence and arousal, respectively).

When means, SDs and sample sizes were not available from the original articles, t- values, F values, or Cohen’s d were also recorded into our data coding. For one study [14], where the author only presented the F value and sample size for arousal rating, we recorded this data to estimate the effect size in the subsequent analysis. When studies presented separate data for multiple experimental stimuli or subscores of rating scale, we computed the effect size for each condition and averaged them. As a result, we only calculated a single effect size for each study. For one study [35], where the experimenter presented participants both food and film and asked them to subjectively rate their feeling, we computed the effect size for each condition first and then combined them as a single mean weighted effect size. When a study further distinguished each group into two different groups and presented separate data (i.e., for patients: those with blunted patients and those without [36]; for healthy controls: participants from America, those from Germany and those from India [37], the means and standard deviations (SDs) were averaged and the averaged means and SDs were used to estimate the final effect sizes.

### Data Analysis

All analyses were performed using the Comprehensive Meta-analysis (CMA) software package [38]. Effect sizes (Cohen’s d) indicate the extent of difference between schizophrenia patients and the healthy controls. Standard meta-analytic methods were employed to compute averaged weighted effect sizes (random model) across studies [39]. For “trait” hedonic studies, positive effect size values mean that patients with schizophrenia had more anhedonia symptoms than healthy controls. For “state” hedonic studies assessing hedonic and exciting experience, positive effect size values mean that patients with schizophrenia had more hedonic or exciting experience than healthy controls. For “state” hedonic studies measuring aversion and calmness experience to positive stimuli, positive effect size values mean that patients with schizophrenia had more aversive or calmness experience than healthy controls. The stability of the mean effect was estimated by its 95% CI. In addition, the homogeneity statistic, Q, was calculated to test whether individual study effect sizes for any given variable reflected a single common population effect size. When the Q statistic approaches significance, it indicates heterogeneity for effect sizes from the individual study. In this case, potential moderators (continuous variable and categorical variable) that accounted for the heterogeneity of effect size were further examined by using mixed effects regression (method of moments) and comparison, respectively. If meta-regression (p value for Z_slope_) or comparison (p value for total Q_Between_) approaches significance, it indicates that the potential moderator might account for the variance of the effect size.

In the current study, the proportion of male patients, estimated negative symptoms, and patient type (inpatient vs. outpatient) were considered as potential moderators to examine the possible variance of effect size in patients with schizophrenia. It should be noted that negative symptoms were rated with two different scales (i.e. either PANSS or SANS) across the studies. Given that the number of items and the scaling are different in these two scales, we had to estimate negative symptoms for each study (negative symptoms scores presented in the studies were divided by the total score of the subscales or items used). For example, in Simon et al,’s study, where PANSS (7 items, 7-points scale) was applied to estimate negative symptoms in schizophrenia patients, the negative subscale score of PANSS (Score = 18.1) was divided by the maximum negative subscale score of PANSS (n = 49). Thus, the estimated negative symptoms score for this study was 0.37. Finally, a fail-safe number which estimates the number of unpublished studies with nil or minimal effect sizes were computed to reduce an overall effect size to some specified negligible value [40], [41]. We set this negligible level at 0.2 and assumed a value of 0.1 for hypothetically “missing” or unpublished studies.

## Results

### Overall Description of Studies

Table S1 and Table S2 summarizes the demographic information and averaged effect sizes of each individual study examining trait and state positive emotional experience, respectively (See details in the supplementary files (Table S1, Table S2 and Reference S1)). There were 47 articles investigating trait hedonic capacity. Eighty-seven percent of the studies (41 of 47) indicated that patients with schizophrenia self-reported more anhedonia symptoms when compared with healthy controls with moderate to large effect sizes (Cohen’ s *d* ≥0.50). There were 44 studies examining state hedonic experience by employing laboratory based measurements. Among these, 40 studies examined valence dimension while 21 studies examined arousal dimension. For the valence studies, 32% of the studies (8 of 25) using bipolar scales and 25% (4 of 16) using unipolar scales showed that patients’ reaction was more anhedonic than healthy controls with a moderate to large effect size (Cohen’s *d*≤−0.50). Besides, patients with schizophrenia subjectively felt more aversive emotion than controls with a moderate to large effect size (Cohen’s *d* ≥0.50) in nearly all studies using unipolar scales (85% of studies (11 of 13)). For arousal, 31% of the studies (5 of 16) using bipolar scales and 20% of the studies (1 of 5) using unipolar scales reported that patients’ reaction to positive stimuli was less than controls with a small effect size (Cohen’s *d* = −0.46 to −0.26). However, both studies using uniploar scales showed that patients’ response was calmer than healthy controls with a small to moderate effect size (Cohen’s *d* = 0.41 and 0.70, respectively).

### Positive Emotional Experience in Schizophrenia vs. Controls

For “trait” studies, the weighted effect size for patients vs. controls is presented in Table 1. Patients with schizophrenia reported significantly more trait anhedonia symptoms than healthy controls with a large effect size (Cohen’s *d* = 0.81). The value of confidence intervals and Orwin statistics suggest that the effect size was stable.

**Table 1:** Mean weighted effect size computed for “trait” and “state” hedonic studies.

Positive Emotion Condition	N _Studies_	N _Patients_	N _Controls_	ES	95% CI	Q value, *p*	Orwin Statistic
**Self report Assessments** a	47	1795	1927	0.81	0.72 to 0.90	113.3, *p*<0.001	279
**Laboratory Based Assessments**							
**Valence**							
Overall (Hedonic Experience)^b^	40	1193	1071	−0.24	−0.37 to −0.112	106.61, *p*<0.001	0
Bipolar Rating^b^	25	672	651	−0.24	−0.41 to −0.07	58.48, *p*<0.001	0
Unipolar Rating							
Hedonic Experience^b^	16	534	446	−0.23	−0.43 to −0.03	48.51, *p*<0.001	0
Aversive Experience^c^	13	392	361	0.75	0.36 to 1.14	115.65, *p*<0.001	56
**Arousal**							
Overall (Exciting Experience)^b^	21	644	614	0.01	−0.14 to 0.16	38.14, *p* = 0.009	0
Bipolar Rating^b^	16	512	482	−0.01	−0.18 to 0.17	24.90, *p* = 0.023	0
Unipolar Rating							
Exciting Experience^b^	5	132	132	0.06	−0.24 to 0.37	10.03, *p* = 0.04	0
Calm Experience^c^	2	56	35	0.56	0.19 to 0.94	0.53, *p* = 0.47	–

***Note:*** ES = Mean Weighted Effect Size;

a positive effect size values mean patients with schizophrenia demonstrate more trait anhedonia symptom than healthy controls.

b positive effect size values mean patients with schizophrenia demonstrate more hedonic or exciting experience than healthy controls.

c positive effect size values mean patients with schizophrenia demonstrate more aversive or calm experience than healthy controls.

We also presented data (overall) regarding with valence and arousal, respectively, which combined studies using either bipolar or unipolar scales to assess hedonic or exciting experience.

For “state” studies, contrary to the findings for “trait” studies, patients with schizophrenia and controls almost did not differ in their overall hedonic experience (valence) to positive stimuli (Cohen’s *d* = −0.24) (See Table 1). We also did not find patients and controls to differ in their hedonic experience when we divided studies based on the type of rating scales (bipolar scales (Cohen’s *d* = −0.24) vs. unipolar scales (Cohen’s *d* = −0.23)). However, patients with schizophrenia reported experiencing more aversion to positive stimuli when compared with healthy controls on unipolar aversion scales with a moderate effect size (Cohen’s *d* = 0.75). For the arousal level, patients with schizophrenia and controls did not differ in their overall arousal experience to positive stimuli (Cohen’s *d* = 0.01) and arousal experience to positive stimuli using both bipolar scales and unipolar scales (Cohen’s *d* = −0.01 and 0.06, respectively). Despite the limited number of studies included in the current analysis, patients with schizophrenia seemed to report more calmness to positive stimuli compared with controls on the unipolar calm scale (Cohen’s *d* = 0.56). The value of confidence intervals and Orwin statistics suggest that the effect size was also stable.

### Moderator Variables

We examined the moderating effect of estimated negative symptoms, the proportion of male patients, and patient type (inpatient vs. outpatient) (See Table 2). Since the number of studies employing unipolar arousal scales was too small to perform a regression analysis, we did not examine the effect of moderator variables of using unipolar scales on state arousal experience.

**Table 2:** Effect of weighted moderators on standard difference in means for “trait” and “state” hedonic studies.

Moderators	Trait	State					
		Valence				Arousal	
		Overall	Bipolar	Unipolar		Overall	Bipolar
				Hedonic	Aversive		
**Estimated Negative Symptom**							
Total N of Studies	26	32	22	11	9	17	13
Z _Slope_	3.36**	0.29	0.79	2.37*	0.90	0.44	−0.70
**% Male of SZ**							
Total N of Studies	44	40	25	16	13	20	15
Z _Slope_	3.16**	2.05*	1.15	1.79^†^	0.20	0.65	0.71
**Patients type**							
Total N of Studies (In. vs. Out.)	8 vs. 21	9 vs. 10	8 vs. 7	1 vs. 3	1 vs. 1	5 vs. 5	5 vs. 4
Q _Between_	0.72	0.74	2.92^†^	0.62	–	0.80	1.03

***Note:*** ^†^: 0.05<*p*≤0.1; *: *p*<0.05; **: *p*<0.01. In. = Inpatients; Out. = Outpatients;

Significanlty positive value of Z_Slope indicates that the value of effect size would get larger with the growing level of moderators.

Significanlty negative value of Z_Slope indicates that the value of effect size would get smaller with the growing level of moderators.

Significant value of Between Q indicates that the level of moderator would contribute to the level of effect size.

Estimated negative symptoms were found to be a significant moderator of the effect size for trait anhedonia (Z _slope_ = 3.36, *p*<0.01, N = 26). The effect size for trait anhedonia increased as the severity of negative symptoms increased. However, negative symptoms did not affect state hedonic and exciting experience in schizophrenia (overall and bipolar rating: all p values for Z _slope_ >0.05). There was only one exception for the moderator effect of negative symptoms on state hedonic assessed by unipolar scale (negative symptoms: Z _slope_ = 2.37, *p* = 0.02, N = 11).

The proportion of male patients was also a significant moderator which contributed to the variation of results in trait studies (Z _slope_ = 3.16, *p*<0.01, N = 44). Samples with more male patients were associated with more trait anhedonia in schizophrenia. Contrary to the finding above, the results in state positive affect revealed that samples with more male patients were associated with less impairment in state hedonic experience in schizophrenia. (Overall Valence: Z _slope_ = 2.05, *p* = 0.04, N = 40). The effect size of state arousal experience was not significantly mediated by gender (All p values >0.05).

Patient type, however, did not contribute to the variation of effect size (all p values of Q _Between_ >0.05).

## Discussion

Our findings demonstrated that patients with schizophrenia reported attenuated trait hedonic capacity with a large effect size in studies using trait self-reported measures. On the other hand, state pleasure (valence) as well as arousal experience were not impaired in schizophrenia patients in response to positive stimuli. Consistent with Cohen and Minor’s meta-analysis [9], patients with schizophrenia showed more state aversion experience to positive stimuli with a moderate effect size. In addition, negative symptoms and the proportion of male patients were significant moderators contributing to the heterogeneity of effect size in trait hedonic capacity in schizophrenia.

### Positive Emotional Experience in Schizophrenia

#### Trait hedonic capacity

In the current meta-analysis, schizophrenia patients had an elevated level of trait anhedonia compared with healthy controls with a large effect size, which is consistent with conclusion stated in prior reviews and meta-analyses [4], [5], [6], [10], [35]. Trait anhedonia is widely accepted as a core deficit and plays an essential role in schizophrenia, especially negative symptoms [1], [2], [10], [35]. Meehl postulated that anhedonia, particularly in the social domain, is a core indicator of vulnerability of schizophrenia and schizotypy [42], [43] and could even predict the development of schizophrenia spectrum disorder [44], [45]. In a recent review, Strauss and Gold indicated that patients with schizophrenia have deficit in their “noncurrent” hedonic capacity assessed by trait, hypothetical, and retrospective measurement [46]. In summary, it is clear that trait hedonic capacity is impaired in patients with schizophrenia which suggested that trait anhedonia may be a reliable marker for the development of next-generation clinical assessment of negative symptoms in schizophrenia [47].

#### State positive emotional experience: valence

In the current analysis, we included an additional 23 studies using either bipolar or unipoar scales into the meta-analysis. In contrast to the study by Cohen and Minor (N = 26) [9]. we did not include nine of the 26 studies in their meta-analysis due to unavailability of full texts of paper or incomplete data for analysis. Consistent with the meta-analysis by Cohen and Minor [9], our findings confirmed that patients with schizophrenia and healthy controls did not differ in state hedonic experience (valence) across studies and it in fact seemed to remain the same irrespective of the kind of scale used (bipolar vs. unipolar). On the other hand, data from the imaging literature suggest a relatively mixed picture [48]. Some studies suggested that schizophrenia patients have no deficit in their neural activities in regions linked to processing positively valenced pictures or money [16], [49]. However, a number of these studies reported that patients with schizophrenia had abnormal neural activation in the amygdala, the hippocampal gyrus, the ventral striatum, the putamen, the insula, and the medial prefrontal cortex while processing positive facial expression [27], [50], [51], [52], IAPS pictures [27], [53], drink [54], [55] and money [56], [57]. Taken together, at least from the perspective of behavioural observation, patients with schizophrenia seem to have no deficit in their state hedonic experience.

In addition, we also found that patients with schizophrenia reported more aversion to positive stimuli which is consistent with the findings in Cohen and Minor’s study. Two possibilities postulated by Cohen and Minor could explain this finding [9]. One is that patients with schizophrenia are impaired in emotional regulation which could lead to disorganization of emotional experience [9]. The other is that patients with schizophrenia had a high level of ambivalence while processing the positively valenced stimuli [9], [58].

#### State positive emotional experience: arousal

Our findings revealed that state arousal experience was also preserved in patients with schizophrenia. Kring [13] suggested that the valence-arousal affective circumplex exists in healthy controls as well as patients with schizophrenia and the pattern of this two-dimensional valence-arousal solution for schizophrenia and controls is similar.

A number of laboratory studies have reported that patients with schizophrenia reported comparable arousal rating relative to healthy sample while processing positive stimuli including pictures from IAPS [20], [59], [60], odour [61], [62], [63], food and film clips [35]. Since arousing stimuli or context would affect cognitive performance (i.e. memory) [64] and feeling state [65], a few studies have examined affective reaction in patients with schizophrenia by further matching condition on emotional arousal. It was found that patients with schizophrenia did not report different arousal experience from healthy controls during the presentation of either high arousing or low arousing positively valenced pictures, words and faces [15], [19], [27]. Consistent with the studies assessing subjective rating, studies examining automatic arousal also reported that patients with schizophrenia displayed comparable physiological response (i.e. skin conductance, startle modulation, breathing rate) while processing positive stimuli [19], [21], [59]. Studies that employed functional imaging, however, provided a relative mixed picture. In one study, Dowd and Barch found that patients with schizophrenia displayed reduced neural activation with positive high arousal condition but not with positive low arousal condition in the left putamen (ROI) when compared with healthy controls. However, such activation interaction (Valence×Arousal) did not appear to be significant in the right ventral striatum between healthy controls and patients. Another imaging study compared cerebral activations in schizophrenia patients and healthy controls during memory retrieval of emotional images that varied in both valence and arousal and revealed that schizophrenia patients closely resemble the control group at both the behavioural and neurofunctional level [66]. Since the number of imaging studies assessing neural response associated with arousal is still small, more studies are needed in the future to elucidate not only whether patients with schizophrenia display different pattern of neural activation linking to arousal compared with healthy samples but also whether and to what extent the neural responses are affected by contributing factor such as negative symptoms, gender, duration of illness, social context and medication.

Taken together, our findings in state arousal (exciting experience), which is similar to those in state valence, further confirmed that state positive emotional experience is not impaired in patients with schizophrenia and anhedonia in schizophrenia might reflect a set of beliefs related to low “noncurrent” pleasure (i.e. trait hedonic capacity) rather than “current” pleasure [46]. Clinical interventions may therefore be developed to improve the processing of “noncurrent” positive affect (i.e. anticipatory, retrospective, trait) rather than “current” positive affect in schizophrenia.

#### Relationships between positive affect and cognitive function

Our findings also support the notion that patients with schizophrenia have no deficit in “in the moment/cosummatory” but problems with “noncurrent” positive affect (i.e. anticipatory pleasure, trait hedonic capacity) which requires more complex cognitive skills, such as imagination/representation, reflection, retrieval of past positive affect, and maintenance of positive emotional state [46], [67]. In recent years, researchers have become aware of the importance of cognitive function in the processing of positive emotion. An increasing number of studies have focused on the link between positive affect and cognitive skills and found that patients with schizophrenia had more difficulties in positive affect which requires imagination/representation [68], [69], consolidation or maintenance of experienced emotion [70], [71], and the retrieval of past positive experience [72]. Taken together, cognitive skills mentioned above play important roles in the processing of positive affect which may also explain the dissociation of trait and state positive experience in schizophrenia [4].

### Moderators

#### Negative symptoms

Our findings revealed that negative symptoms could moderate the effect size of “trait” hedonic capacity (p<0.01). In other words, samples with elevated level of negative symptoms were associated with more trait anhedonia in schizophrenia. The present finding is consistent with prior studies which examined trait anhedonia in schizophrenia. Horan (2003) found that patients with the deficit syndrome of schizophrenia reported more social anhedonia than patients without the deficit syndrome [23]. Suslow et al. further showed that anhedonic schizophrenia patients demonstrated more physical and social anhedonia than other patients or healthy groups [24], [73]. Chan et al. also found that schizophrenia patients with negative symptoms reported more impairment in anticipatory pleasure than patients without negative symptoms [74].

For studies looking at “state” positive affect, our findings did not support that negative symptoms could contribute to the variance of findings in terms of valance and arousal. Some previous studies also did not find significant relationships between negative symptoms and subjective emotional experience rating [15], [75], [76] or physiological response to positive stimuli [16], [20]. In two studies, patients with schizophrenia were further divided into two subgroups (blunted vs. non-blunted or deficit vs. non-deficit) and the findings revealed no significant difference between these two sub-groups while processing positive film clips or drink [36], [77]. However, other studies revealed that negative symptoms in schizophrenia are inversely correlated with their state hedonic experience concerning valence [27], [35] and neural response (e.g. left ventral striatum and amygdale) in schizophrenia [27], [48], [78] which are not consistent with our findings.

It is surprising to note that patients with more severe negative symptoms had less severe deficit in state hedonic experience (Z_slope_ = 2.37, p<0.05). To our knowledge, no study has reported this finding. One possibility is that the odd finding might be related to the type of scale used to assess positive affect. In the current analysis, all the studies included employed unipolar scales. Similarly, analysis for gender effect revealed a different finding in studies using unipolar scales. However, it is unclear whether the postulation is true or not. Due to the small number of studies (N = 11) included into the regression model, it may be premature to draw a conclusive remark on these findings.

Taken together, negative symptoms mediate positive affect, especially trait hedonic capacity in schizophrenia. However, our finding did not support an important role of negative symptoms in state positive affect. For clinical practice, since the presence of negative symptoms is linked to poor community and social functioning [70], [79], the development of intervention which aims at improving anhedonia might in turn alleviate the severity of negative symptoms and enhance quality of life and outcome in patients with schizophrenia.

#### Gender effect

The current findings showed that samples with more male patients were associated with more trait anhedonia in schizophrenia and are consistent with Kring and Moran’s claims of a gender difference [10]. However, the number of prior studies investigating the effect of gender on trait anhedonia in schizophrenia is relatively small [28].

For studies on “state” hedonic capacity, the findings provided a mixed picture in valence and arousal. Our current results showed that more male patients were unexpectedly associated with less impairment in positive affect (valence) in schizophrenia, and gender did not moderate the effect size of arousal in schizophrenia. These findings are partially consistent with those suggested by a prior meta-analysis [9]. Cohen and Minor compared effect sizes of studies employing primarily male and mixed gender patients and did not find that gender effect would affect emotional response (mainly valence) while processing positive stimuli [9]. Similarly, several prior studies [60], [62], [70], [80] except one [30] specifically examining the influence of gender on positive affect (valence or arousal) also did not report a positive finding. These unexpected findings for valence may be due the different methodologies adopted by different studies. In Cohen and Minor’s study, they compared the effect sizes between studies employing primarily male (defined as sample as >90% male) and studies employing mixed gender. In our study, we performed a regression model in which percentages of male patients was regarded as a predictor variable. It is possible that the latter approach is more sensitive. There might also be a possibility that the number of studies enrolling high percentage of female patients was exceptionally small. Since samples with higher percentages of female patients might also be associated with less difficulties in emotional processing [10], increasing the number of studies enrolling high percentages of female patients might reduce the degree of association between gender and state positive affect. Thirdly, is the difference may be related to the type of scales used to assess positive affect as mentioned above.

Taken together, our findings suggest that female patients with schizophrenia are less deficient in trait hedonic capacity compared to male patients. The number of studies enrolling high percentages of female patients to specifically examine how gender affect state positive affect is still limited and no conclusive remark could be drawn on this issue. Further study is needed to address whether and how gender affects state positive emotional processing in schizophrenia.

#### Other confounding factors

Other confounding factors such as patient type, medication status, illness course, and personality trait are discussed here. In this study, no evidence was found for patient type (inpatient vs. outpatient) as a confounding factor for trait or state positive affect in schizophrenia which is consistent with prior meta-analytic findings examining emotional perception [81]. It is suggested that inpatients and outpatients with schizophrenia share similar pattern in the processing of trait and state positive affect. For medication status, since most studies recruited patients who were on medication, we did not examine the effect of medication status in this study. Prior reviews have suggested that medication status might not contribute to the variance of findings in emotional processing in schizophrenia [9], [82]. For illness course, we additionally performed a set of regression analysis to test whether duration of illness would contribute to the variance of findings. No significant result was found. Lastly, it should also be of note that trait anhedonia/low positive affect in schizophrenia might also be related to personality trait (extraversion) [5], elevated level of current depressive symptom [16], [83] and quality of life [84].

### Limitations

The current meta-analysis has a number of limitations. First, for emotional arousal, we have limited the scope of our study to positive affect. In future, studies should also examine arousal experience in response to negative and neutral stimuli because some studies had reported that patients with schizophrenia showed a different pattern in the processing of neutral and negative stimuli [85], [86]. Second, the number of laboratory studies included in the current analysis examining arousal experience using unipolar scales was relatively small. Our findings should be viewed as preliminary and interpreted with caution. Lastly, the scope of the current meta-analysis is limited to behavioural and self-reported data. With increasing knowledge of neural mechanisms associated with hedonic experience in schizophrenia, it would be of interest to elucidate whether or not neural responses linked to hedonic experience is normal in schizophrenia. Future study should also examine the neural basis associated with hedonic affect in schizophrenia.

In conclusion, the current study provides important evidence to show that patients with schizophrenia do not exhibit deficits in their state positive affective experience including both valence and arousal. However, robust findings have shown that patients with schizophrenia exhibit deficits in their “noncurrent” positive affect (i.e. trait hedonic capacity). Gender and negative symptoms also partially influence the reaction to positive affect (especially trait hedonic capacity) in patients with schizophrenia. Our findings suggest that it may be useful for researchers to explore the neural activities associated with positive affect in patients with schizophrenia. These findings also provide a potential theoretical basis for future treatment focusing on “noncurrent” positive affect to alleviate negative symptoms as well as to enhance the quality of life in this group of patients.

## Supporting Information

Table S1 **Descriptive information, effect size and variance score computed for studies assessing “trait” hedonic capacity.**
(DOC)

Table S2 **Descriptive information, effect size and variance score computed for studies examining “state” positive affect.**
(DOC)

Reference S1 **References list containing studies examining either trait or state positive affect included in the meta-analysis.**
(DOC)
