# Supplementary material for: Trait and State Positive Emotional Experience in Schizophrenia: A Meta-Analysis
Source: PLoS One. 2012 Jul 18;7(7):e40672. doi: 10.1371/journal.pone.0040672 (PMC3399884; doi:10.1371/journal.pone.0040672)
Supplement: Table S1 — Descriptive information, effect size and variance score computed for studies assessing “trait” hedonic capacity. (DOC) [file pone.0040672.s001.doc]

**Table S1 Descriptive information, effect size and variance score computed for studies assessing “trait” hedonic capacity**

**Patients vs. Healthy Controls: “Trait” hedonic studies (N = 47)**

| **Articles** | **Scales** | **Medicated** | **N _SZ-HC_** | **% _Male_** | **Course** | **NS** | **ES ± VAR** |
| --- | --- | --- | --- | --- | --- | --- | --- |
| Katsanis et al., 1990 | CPAS, CSAS | No | 38-160 | - | FEP | - | 0.98 ± 0.02 |
| Clementz et al., 1991 | CPAS | - | 54-178 | 53 | Both | - | 1.04 ± 0.03 |
| Grove et al., 1991 | CPAS | - | 17-18 | 63 | Chronic | - | 1.00 ± 0.13 |
| Schlenker et al.,1995 | Mix. (CPAS, CSAS) | Both | 34-24 | 100 | Chronic | 0.26 | 1.63 ± 0.09 |
| Heerlein et al., 1996 | MP-T | - | 16-21 | 65 | - | - | 0.00 ± 0.11 |
| Berlin et al.,1998 | CPAS, CSAS, FCPS | Yes | 20-20 | 45 | Chronic | 0.61 | 0.81 ± 0.04 |
| Blanchard et al.,1998 | CPAS, CSAS | Yes | 37-15 | 67 | Chronic | - | 0.92 ± 0.05 |
| Lubin et al.,1998 | MAACL-R | - | 51-185 | - | - | - | 0.56 ± 0.03 |
| Craver et al., 1999 | CSAS | Both | 39-38 | 56 | Both | 0.275 | 1.08 ± 0.06 |
| Loas et al.,1999 | CPAS, FCPS-PP | Yes | 32-35 | 51 | Chronic | - | 0.76 ± 0.03 |
| Laurent et al., 2000 | CPAS, CSAS | Yes | 23-34 | 77 | Chronic | 0.49 | 1.26 ± 0.06 |
| Horan et al.,2003 a | GTS | Yes | 36-15 | 100 | Chronic | - | 0.69 ± 0.10 |
| Horan et al.,2003 b | CSAS, GTS | Yes | 45-41 | 84 | Chronic | - | 0.88 ± 0.03 |
| Lysaker et al., 2003 | NEO-Ex | Yes | 59-17 | 97 | Chronic | 0.34 | 0.70 ± 0.08 |
| SchuÜrhoff et al.,2003 | CPAS | Yes | 80-94 | 56 | Chronic | - | 0.52 ± 0.02 |
| Suslow et al., 2003 a | CPAS, CSAS | Yes | 88-30 | 54 | Chronic | 0.26 | 0.64 ± 0.02 |
| Suslow et al., 2003 b | CPAS, CSAS | Yes | 88-30 | 54 | Chronic | 0.26 | 0.66 ± 0.02 |
| Anfred et al.,, 2004 | CPAS, CSAS | Yes | 12-14 | 100 | Chronic | 0.40 | 1.35 ± 0.10 |
| Camisa et al., 2005 | CSAS | Both | 63-55 | 82 | Chronic | - | 1.49 ± 0.04 |
| Onisuka et al., 2005 | NEO-Ex | Yes | 24-26 | 100 | Chronic | - | 0.87 ± 0.09 |
| Herrán et al., 2006 | EPQ-Ex | Both | 62-43 | 54 | Chronic | 0.33 | 0.50 ± 0.04 |
| Horan et al.,2006 | CPAS, CSAS | Yes | 30-31 | 75 | Chronic | 0.40 | 1.18 ± 0.04 |
| Burbridge et al.,2007 | CPAS, CSAS | Yes | 49-47 | 56 | Chronic | - | 0.54 ± 0.01 |
| Gard et al.,2007 | TEPS, *CPAS, CSAS* | Yes | 51-50 | 57 | - | - | 0.44 ± 0.02 |
| Barch et al., 2008 | CPAS, CSAS, EPQ-Ex | Yes | 44-66 | 65 | - | 0.36 | 0.80 ± 0.01 |
| Falkenberg et al., 2008 | EPQ-Ex | Yes | 17-17 | 65 | FEP | 0.32 | 0.81 ± 0.13 |
| Herbener et al.,2008 | CPAS, CSAS | Both | 34-35 | 54 | - | - | 0.61 ± 0.03 |
| Waltz et al., 2008 | CPAS, CSAS | Yes | 18-18 | 75 | Chronic | 0.19 | 0.24 ± 0.06 |
| Baslet et al.,2009 | CPAS, CSAS | Both | 21-20 | 68 | Chronic | - | 0.35 ± 0.05 |
| Harvey et al.,2009 | CSAS | Yes | 27-27 | 52 | - | 0.14 | 0.60 ± 0.08 |
| Park et al., 2009 a | CPAS, CSAS | Yes | 27-27 | 52 | - | 0.41 | 1.12 ± 0.04 |
| Park et al., 2009 b | CPAS, CSAS | Yes | 29-21 | 52 | Chronic | 0.34 | 0.92 ± 0.04 |
| Trémeau et al.,2009 | CPAS, CSAS | Yes | 64-32 | 83 | Chronic | 0.50 | 1.14 ± 0.03 |
| Walter et al., 2009 | PAS, SAS | Yes | 16-16 | 47 | Chronic | 0.45 | 0.92 ± 0.08 |
| Cochrane et al., 2010 | O-LIFE | Yes | 20-38 | 71 | - | - | 0.99 ± 0.09 |
| Dowd et al.,2010 | CPAS, CSAS | Yes | 40-32 | 65 | Chronic | 0.54 | 1.19 ± 0.03 |
| Harvey et al.,2010 | CPAS | Yes | 30-26 | 57 | Chronic | 0.15 | 0.08 ± 0.07 |
| Horan et al.,2010 | CPAS, CSAS | Yes | 38-36 | 78 | Chronic | 0.46 | 1.21 ± 0.03 |
| Larguet et al.,2010 | CPAS, CSAS | Yes | 21-20 | - | Chronic | - | 1.26 ± 0.06 |
| Simon et al.,2010 | CPAS, CSAS | Yes | 15-15 | 67 | Chronic | 0.37 | 0.19 ± 0.07 |
| Tso et al., 2010 | CPAS, CSAS, AIM, DES | Yes | 33-33 | 68 | Chronic | - | 0.87 ± 0.02 |
| Waltz et al.,2010 | CPAS, CSAS | Yes | 17-17 | 74 | Chronic | 0.24 | 0.48 ± 0.06 |
| Wynn et al., 2010 | CPAS, CSAS, TEPS | Yes | 34-36 | 73 | Chronic | 0.48 | 0.81 ± 0.02 |
| Becerril et al.,2011 | CPAS, CSAS | Yes | 38-32 | 66 | Chronic | - | 1.00 ± 0.03 |
| Lee et al.,2011 | CPAS | Yes | 46-56 | 55 | Chronic | 0.31 | 0.77 ± 0.04 |
| Strauss et al., 2011a | PANAS | Yes | 32-27 | 64 | - | 0.30 | 0.54 ± 0.07 |
| Strauss et al., 2011b | TEPS, CPAS, CSAS | Yes | 86-59 | 62 | - | - | 0.71 ± 0.01 |

***Note*:** “Both” = Medicated + Naïve or FEP + Chronic; FEP = First episode patients; ES = Effect size; VAR = Variance score; SZ = Schizophrenia patients; HC = Healthy controls; NS = Estimated Negative Symptom; positive effect size value indicate that patients demonstrate more anhedonia than controls. CPAS = Chapman Physical Anhedonia Scale; CSAS = Chapman Social Anhedonia Scale; FCPS = Fawcett Clark Pleasure Scale; TEPS = Temporal Experience of Pleasure Scale; GTS = General Temperament Surveys; EPQ-EX = Eysenck Personality Questionnaire -Extraversion subscale; O-LIFE = Oxford-Liverpool Inventory of Feelings and Experiences; MP-T = Von Zerssen’s Munich Personality Test; NEO-Ex = NEO Five Factors Inventory-Extraversion; AIM = Affect Intensity Measure; DES = the Differential Emotion Scale; PANAS = Positive and Negative Affect Scale. The scales which are typed in italic mean that the data for these scales were not available. “Mix.” indicates that the study report combined data for both CPAS and CSAS.
