# Supplementary material for: Trait and State Positive Emotional Experience in Schizophrenia: A Meta-Analysis
Source: PLoS One. 2012 Jul 18;7(7):e40672. doi: 10.1371/journal.pone.0040672 (PMC3399884; doi:10.1371/journal.pone.0040672)
Supplement: Table S2 — Descriptive information, effect size and variance score computed for studies examining “state” positive affect. (DOC) [file pone.0040672.s002.doc]

**Table S2 Descriptive information, effect size and variance score computed for studies examining “state” positive affect**

**Patients vs. Healthy Controls: “State” hedonic Studies (Valence (N = 40) vs. Arousal (N = 21))**

| **Articles** | **Stimuli** | **Medicated** | **N _SZ-HC_** | **% _Male_** | **Course** | **NS** | **Scales** | **ES ± VAR** | | | |
| --- | --- | --- | --- | --- | --- | --- | --- | --- | --- | --- | --- |
|  |  |  |  |  |  |  |  | **Valence** | | **Arousal** | |
|  |  |  |  |  |  |  |  | **Hedonic** | **Aversive** | **Exciting** | **Calm** |
| Berenbaum et al.,1992 ^a^ | Drink and Film | Yes | 43-20 | 51 | Chronic | - | Unipolar | 0.20 ± 0.04 | N/A |  |  |
| Schlenker et al.,1995 ^a^ | Picts | Both | 34-24 | 100 | Chronic | 0.26 | Bipolar | -0.12 ± 0.07 |  | 0.22 ± 0.07 |  |
| Schneider et al.,1995 ^a^ | Picts | Both | 40-40 | - | Chronic | 0.34 | Unipolar | -0.25 ± 0.03 | 0.60 ± 0.03 |  |  |
| Kring et al.,1996 ^a^ | Film | No | 23-20 | 100 | Chronic | - | Unipolar | 0.13 ± 0.09 | 0.97 ± 0.10 |  |  |
| Schneider et al.,1998 ^a^ | Picts | Yes | 13-13 | 100 | Chronic | 0.28 | Unipolar | -0.26 ± 0.16 | 1.36 ± 0.19 |  |  |
| Earnst et al.,1999 ^b^ | Film | Yes | 41-20 | 100 | Chronic | - | Unipolar | -0.26 ± 0.07 | 0.90 ± 0.08 | 0.18 ± 0.08 | 0.41 ± 0.08 |
| Kring et al.,1999 ^a^ | Film | Both | 15-15 | 100 | Chronic | 0.39 | Unipolar | 0.04 ± 0.07 | 0.75 ± 0.07 | 0.55 ± 0.07 | 0.70 ± 0.07 |
| Salem et al.,1999 ^a^ | Film | Yes | 17-15 | 100 | Chronic | 0.46 | Unipolar | -0.63 ± 0.13 | 0.86 ± 0.14 |  |  |
| Habel et al.,2000 ^a^ | Face | Both | 93-93 | - | Chronic | - | Unipolar | -0.41 ± 0.02 | -0.56 ± 0.02 |  |  |
| Crespo-Facorro et al.,2001 ^a^ | Odorant | No | 18-16 | 68 | Both | - | Bipolar | -0.65 ± 0.12 |  | 0.20 ± 0.12 |  |
| Hurdry et al.,2002 ^b^ | Odorant | Yes | 40-40 | 50 | Chronic | 0.58 | Bipolar |  |  | -0.14 ± 0.05 |  |
| An et al., 2003 ^a^ | Face | Both | 20-20 | 58 | Chronic | 0.33 | Bipolar | 0.15 ± 0.10 |  |  |  |
| Paradiso et al.,2003 ^a^ | Picts | No | 18-17 | 66 | Both | - | Bipolar | -0.83 ± 0.12 |  |  |  |
| Volz et al.,2003 ^a^ | Picts | Both | 49-46 | 42 | Chronic | 0.17 | Bipolar | -0.28 ± 0.05 |  | 0.34 ± 0.05 |  |
| Habel et al.,2004 ^a^ | Face | Yes | 13-26 | 100 | Chronic | 0.37 | Unipolar | -0.04 ± 0.12 | 0.52 ± 0.12 |  |  |
| Mathews et al.,2004 ^b^ | Words | N/A | 27-28 | 64 | Chronic | 0.33 | Bipolar | -0.51 ± 0.04 |  | -0.37 ± 0.04 |  |
| Hempel et al.,2005 ^b^ | Picts | Both | 28-30 | 81 | - | 0.41 | Bipolar | -0.09 ± 0.07 |  | -0.29 ± 0.07 |  |
| Holt et al.,2005 ^b^ | Face | Yes | 18-16 | 100 | Chronic | 0.35 | Bipolar | 0.15 ± 0.12 |  |  |  |
| Rupp et al.,2005 ^b^ | Odorant | Both | 33-40 | 100 | - | 0.42 | Both | 0.27 ± 0.03 |  | -0.38 ± 0.03 | N/A |
| Taylor et al.,2005 ^b^ | Picts | Both | 18-10 | 61 | Chronic | 0.34 | Bipolar | -1.06 ± 0.18 |  |  |  |
| An et al., 2006 ^b^ | Picts | Yes | 20-22 | 48 | Chronic | 0.25 | Bipolar | 0.00 ± 0.10 |  | -0.10 ± 0.10 |  |
| Habel et al.,2006 ^a^ | Face | No | 20-20 | 50 | Chronic | - | Unipolar | -0.77 ± 0.04 | 1.83 ± 0.03 |  |  |
| Horan et al.,2006 ^a^ | Food and Film | Yes | 30-31 | 75 | Chronic | 0.40 | Unipolar | -0.34 ± 0.03 | 0.56 ± 0.03 | 0.01 ± 0.03 | N/A |
| Lee et al.,2006 ^a^ | Picts | Yes | 21-20 | 44 | Chronic | 0.25 | Bipolar | 0.13 ± 0.10 |  |  |  |
| Rockstroh et al.,2006 ^b^ | Picts | Yes | 12-12 | 100 | Chronic | 0.40 | Bipolar | 0.31 ± 0.18 |  | 0.43 ± 0.18 |  |
| Seok et al.,2006 ^a^ | Picts and Words* | Yes | 25-25 | 52 | - | 0.33 | Bipolar | -0.86 ± 0.09 |  |  |  |
| Burbridge et al.,2007 ^a^ | Sound, Picts, Words, Film* | Yes | 49-47 | 56 | Chronic | - | Bipolar |  |  | -0.26 ± 0.04 |  |
| Hempel et al.,2007 ^b^ | Picts | Both | 26-21 | 100 | FEP | - | Bipolar | -0.06 ± 0.04 |  | 0.27 ± 0.04 |  |
| Henry et al.,2007 ^a^ | Film | Yes | 29-30 | 73 | Chronic | 0.31 | Unipolar | -0.54 ± 0.07 | N/A |  |  |
| Reske et al.,2007 ^b^ | Imitation | Yes | 10-10 | 60 | FEP | 0.30 | Unipolar | -1.19 ± 0.06 | 0.44 ± 0.05 |  |  |
| Schneider et al.,2007 ^b^ | Odorant | Both | 13-26 | 100 | Chronic | 0.37 | Bipolar | -0.09 ± 0.12 |  |  |  |
|  |  |  |  |  |  |  | Unipolar | 0.19 ± 0.06 | 0.79 ± 0.04 | 0.05 ± 0.06 | N/A |
| Park et al., 2009 ^a^ | Social Interaction | Yes | 27-27 | 52 | - | 0.41 | Bipolar | -0.09 ± 0.07 |  |  |  |
| Trémeau et al.,2009 ^a^ | Sound, Picts, Words* | Yes | 64-32 | 84 | Chronic | 0.50 | Unipolar | 0.27 ± 0.05 | 0.90 ± 0.05 |  |  |
| Dowd et al., 2010 ^a^ | Picts, Words, Face* | Yes | 40-32 | 65 | Chronic | 0.54 | Bipolar | -0.84 ± 0.03 |  |  |  |
| Mathews et al.,2010 ^a^ | Face, Pict* | Yes | 40-40 | 64 | Chronic | 0.29 | Bipolar | -0.85 ± 0.05 |  |  |  |
| Rauch et al., 2010 | Face | Yes | 12-12 | 33 | - | 0.39 | Bipolar | -0.57 ± 0.17 |  |  |  |
| Seubert et al., 2010 ^b^ | Odorant | Both | 24-24 | 60 | - | 0.31 | Bipolar | -0.22 ± 0.09 |  | 0.00 ± 0.09 |  |
| Simon et al.,2010 ^a^ | Monetary | Yes | 15-15 | 67 | Chronic | 0.37 | Bipolar | 0.13 ± 0.13 |  | 0.99 ± 0.15 |  |
| Trémeau et al.,2010 ^a^ | Sound, Picts* | Yes | 70-35 | 83 | Chronic | 0.48 | Unipolar | 0.10 ± 0.02 | N/A |  |  |
| Wynn et al., 2010 ^a^ | Picts | Yes | 34-36 | 73 | Chronic | 0.48 | Bipolar |  |  | 0.13 ± 0.06 |  |
| Yee et al.,2010 ^b^ | Picts | Yes | 77-74 | 68 | Combined | 0.37 | Bipolar | -0.01 ± 0.03 |  | -0.39 ± 0.03 |  |
| Gard et al., 2011 ^a^ | Picts | Yes | 28-19 | 66 | - | 0.35 | Bipolar |  |  | 0.22 ± 0.09 |  |
| Kring et al.,2011 ^a^ | Picts | Yes | 31-28 | 64 | Chronic | 0.35 | Bipolar | -0.40 ± 0.07 |  | -0.46 ± 0.07 |  |
| Lepage et al.,2011 ^b^ | Face | Yes | 26-26 | 52 | - | 0.22 | Bipolar | 0.57 ± 0.15 |  |  |  |

***Note*:** ^a^ Task introduction involved having participants asked to rate their subjective experience to the positive stimuli; ^b^ Task introduction involved having participants rate the stimuli itself; * refers that studies reported merged data of response to stimuli from multiple modality; “Both” = Medicated + Naïve or FEP + Chronic or Bipolar + Unipolar; FEP = First episode patients; ES = Effect size; VAR = Variance score; SZ = Schizophrenia patients; HC = Healthy controls; NS = Estimated Negative Symptom; positive effect size value from bipolar and unipolar hedonic valence ratings indicate that patients report more hedonic experience than controls; positive effect size value from unipolar aversive ratings indicate that patients report more negative affective experience than controls; positive effect size value from bipolar and unipolar exciting arousal ratings indicate that patients report more excitement than controls; positive effect size value from unipolar calm ratings indicate that patients report more calmness than controls. Unipolar scale refers to subjective rating scale assessing either state hedonic/exciting or aversive/calm emotion in separate scales, whereas bipolar scale refers to subjective rating scale set up with both extreme state hedonic/exciting and aversive/calm emotion on opposing ends of a continuum.
