# Supplementary material for: Trait and State Positive Emotional Experience in Schizophrenia: A Meta-Analysis
Source: PLoS One. 2012 Jul 18;7(7):e40672. doi: 10.1371/journal.pone.0040672 (PMC3399884; doi:10.1371/journal.pone.0040672)
Supplement: Reference S1 — References list containing studies examining either trait or state positive affect included in the meta-analysis. (DOC) [file pone.0040672.s003.doc]

**Reference S1. References list containing studies examining either trait or state positive affect included in the meta-analysis.**

An SK, Lee E, Kim JJ, Namkoong K, Kang JI, et al. (2006) Greater impairment in negative emotion evaluation ability in patients with paranoid schizophrenia. Yonsei Medical Journal 47: 343-353.

Arnfred SM, Chen ACN (2004) Exploration of somatosensory P50 gating in schizophrenia spectrum patients: reduced P50 amplitude correlates to social anhedonia. Psychiatry Res 125: 147-160.

An SK, Lee SJ, Lee CH, Cho HS, Lee PG, et al. (2003) Reduced P3 amplitudes by negative facial emotional photographs in schizophrenia. Schizophr Res 64: 125-135.

Becerril KK, Barch DD (2011) Influence of emotional processing on working memory in schizophrenia. Schizophrenia Bulletin 37: 1027-1038.

Barch DM (2008) Emotion, motivation, and reward processing in schizophrenia spectrum disorders: What we know and where we need to go. Schizophr Bull 34: 816-818.

Baslet G, Termini L, Herbener E (2009) Deficits in emotional awareness in schizophrenia and their relationship with other measures of functioning. The Journal of Nervous and Mental Disease 197: 655-660.

Berenbaum H, Oltmanns TF (1992) Emotional experience and expression in schizophrenia and depression. J Abnorm Psychol 101: 37-44.

Berlin I, Givry-Steiner L, Lecrubier Y, Puech AJ (1998) Measures of anhedonia and hedonic responses to sucrose in depressive and schizophrenic patients in comparison with healthy subjects. European Psychiatry : The Journal of The Association of European Psychiatrists 13: 303-309.

Blanchard JJ, Mueser KT, Bellack AS (1998) Anhedonia, positive and negative affect, and social functioning in schizophrenia. Schizophr Bull 24: 413-424.

Burbridge JA, Barch DM (2007) Anhedonia and the experience of emotion in individuals with schizophrenia. J Abnorm Psychol 116: 30-42.

Camisa KM, Bockbrader MA, Lysaker P, Rae LL, Brenner CA, et al. (2005) Personality traits in schizophrenia and related personality disorders. Psychiatry Res 133: 23-33.

Clementz BA, Grove WM, Katsanis J, Iacono WG (1991) Psychometric detection of schizotypy: Perceptual aberration and physical anhedonia in relatives of schizophrenics. J Abnorm Psychol 100: 607-612.

Craver JC, Pogite-Qeile MF (1999) Familial liability to schizophrenia: A sibling study of negative symptoms. Schizophr Bull 25: 827-839.

Crespo-Facorro B, Paradiso S, Andreasen NC, O'Leary DS, Watkins GL, et al. (2001) Neural mechanisms of anhedonia in schizophrenia: a PET study of response to unpleasant and pleasant odors. JAMA 286: 427-435.

Dowd EC, Barch DM (2010) Anhedonia and emotional experience in schizophrenia: Neural and behavioral indicators. Biol Psychiatry 67: 902-911.

Earnst KS, Kring AM (1999) Emotional responding in deficit and non-deficit schizophrenia. Psychiatry Res 88: 191-207.

Falkenberg I, Bartels M, Wild B (2008) Keep smiling!: Facial reactions to emotional stimuli and their relationship to emotional contagion in patients with schizophrenia. European Archives of Psychiatry and Clinical Neuroscience 258: 245-253.

Gard DE, Kring AM, Gard MG, Horan WP, Green MF (2007) Anhedonia in schizophrenia: Distinctions between anticipatory and consummatory pleasure. Schizophr Res 93: 253-260.

Gard DE, Cooper S, Fisher M, Genevsky A, Mikels JA, et al. (2011) Evidence for an emotion maintenance deficit in schizophrenia. Psychiatry Research 187: 24-29.

Grove WM, Lebow BS, Clementz BA, Cerri A, Medus C, et al. (1991) Familial prevalence and coaggregation of schizotypy indicators: a multitrait family study. J Abnorm Psychol 100: 115-121.

Habel U, Gur RC, Mandal MK, Salloum JB, Gur RE, et al. (2000) Emotional processing in schizophrenia across cultures: standardized measures of discrimination and experience. Schizophr Res 42: 57-66.

Habel U, Klein M, Shah NO, Toni I, Zilles K, et al. (2004) Genetic load on amygdala hypofunction during sadness in nonaffected brothers of schizophrenia patients. Am J Psychiat 161: 1806-1813.

Habel U, Krasenbrink I, Bowi U, Ott G, Schneider F (2006) A special role of negative emotion in children and adolescents with schizophrenia and other psychoses. Psychiatry Res 145: 9-19.

Harvey P-O, Armony J, Malla A, Lepage M (2010) Functional neural substrates of self-reported physical anhedonia in non-clinical individuals and in patients with schizophrenia. J Psychiatr Res 44: 707-716.

Heerlein A, Santander J, Richter P (1996) Premorbid personality aspects in mood and schizophrenic disorders. Comprehensive Psychiatry 37: 430-434.

Hempel RJ, Tulen JHM, van Beveren NJM, Mulder PGH, Hengeveld MW (2007) Subjective and physiological responses to emotion-eliciting pictures in male schizophrenic patients. Int J Psychophysiol 64: 174-183.

Henry JD, Green MJ, de Lucia A, Restuccia C, McDonald S, et al. (2007) Emotion dysregulation in schizophrenia: Reduced amplification of emotional expression is associated with emotional blunting. Schizophr Res 95: 197-204.

Herbener ES, Song W, Khine TT, Sweeney JA (2008) What aspects of emotional functioning are impaired in schizophrenia? Schizophr Res 98: 239-246.

Herran A, Sierra-Biddle D, Cuesta MJ, Sandoya M, Vazquez-Barquero JL (2006) Can personality traits help us explain disability in chronic schizophrenia? Psychiatry Clin Neurosci 60: 538-545.

Holt DJ, Weiss AP, Rauch SL, Wright CI, Zalesak M, et al. (2005) Sustained activation of the hippocampus in response to fearful faces in schizophrenia. Biol Psychiatry 57: 1011-1019.

Horan WP, Blanchard JJ (2003) Neurocognitive, social, and emotional dysfunction in deficit syndrome schizophrenia. Schizophr Res 65: 125-137.

Horan WP, Blanchard JJ (2003) Emotional responses to psychosocial stress in schizophrenia: the role of individual differences in affective traits and coping. Schizophr Res 60: 271-283.

Horan WP, Green MF, Kring AM, Nuechterlein KH (2006) Does anhedonia in schizophrenia reflect faulty memory for subjectively experienced emotions? J Abnorm Psychol 115: 496-508.

Horan WP, Wynn JK, Kring AM, Simons RF, Green MF (2010) Electrophysiological correlates of emotional responding in schizophrenia. J Abnorm Psychol 2010, 119: 18-30.

Hudry J, Saoud M, d'Amato T, Daléry J, Royet J-P (2002) Ratings of different olfactory judgements in schizophrenia. Chemical Senses 27: 407-416.

Katsanis J, Iacono WG, Beiser M (1990) Anhedonia and perceptual aberration in first-episode psychotic patients and their relatives. J Abnorm Psychol 99: 202-206.

Kring AM, Earnst KS (1999) Stability of emotional responding in schizophrenia. Behavior Therapy 30: 373-388.

Kring AM, Neale JM (1996) Do schizophrenic patients show a disjunctive relationship among expressive, experiential, and psychophysiological components of emotion? J Abnorm Psychol 105: 249-257.

Kring AM, Gard MG, Gard DE (2011) Emotion Deficits in Schizophrenia: Timing Matters. Journal of Abnormal Psychology 120: 79-87.

Larquet M, Coricelli G, Opolczynski G, Thibaut F (2010) Impaired decision making in schizophrenia and orbitofrontal cortex lesion patients. Schizophr Res 116: 266-273.

Laurent A, Biloa-Tang M, Bougerol T, Duly D, Anchisi A-M, et al. (2000) Executive/attentional performance and measures of schizotypy in patients with schizophrenia and in their nonpsychotic first-degree relatives. Schizophr Res 46: 269-283.

Lee E, Kim JJ, Namkoong K, An SK, Seok JH, et al. (2006) Aberrantly flattened responsivity to emotional pictures in paranoid schizophrenia. Psychiatry Res 143: 135-145.

Lee JS, Park HJ, Chun JW, Seok JH, Park IH, et al. (2011) Neuroanatomical correlates of trait anhedonia in patients with schizophrenia: A voxel-based morphometric study. Neuroscience Letters 489: 110-114.

Lepage M, Sergerie K, Benoit A, Czechowska Y, Dickie E, et al. (2011) Emotional face processing and flat affect in schizophrenia: functional and structural neural correlates. Psychological Medicine 41: 1833-1844.

Loas G, Boyer P, Legrand A (1999) Anhedonia in the deficit syndrome of schizophrenia. Psychopathology 32: 207-219.

Lubin B, Van Whitlock R, Zuckerman M (1998) Affect traits in differential diagnosis of anxiety, depressive, and schizophrenic disorders using the multiple affect adjective check list - revised. Assessment 5: 309-319.

Lysaker PH, Wilt MA, Plascak-Hallberg CD, Brenner CA, Clements CA (2003) Personality dimensions in schizophrenia: Associations with symptoms and coping. Journal of Nervous and Mental Disease 191: 80-86.

Mathews JR, Barch DM (2004) Episodic memory for emotional and nonemotional words in schizophrenia. Cognition & Emotion 18: 721-740.

Mathews JR, Barch DM (2010) Emotion Responsivity, Social Cognition, and Functional Outcome in Schizophrenia. J Abnorm Psychol 119: 50-59.

Onitsuka T, Nestor PG, Gurrera RJ, Shenton ME, Kasai K, et al. (2005) Association between reduced extraversion and right posterior fusiform gyrus gray matter reduction in chronic schizophrenia. Am J Psychiatry 162: 599-601.

Paradiso S, Andreasen NC, Crespo-Facorro B, O'Leary DS, Watkins GL, et al. (2003) Emotions in unmedicated patients with schizophrenia during evaluation with positron emission tomography. Am J Psychiat 160: 1775-1783.

Park IH, Kim JJ, Ku J, Jang HJ, Park SH, et al. (2009) Characteristics of social anxiety from virtual interpersonal interactions in patients with schizophrenia. Psychiatry 72: 79-93.

Park IH, Kim J-J, Chun J, Jung YC, Seok JH, et al. (2009) Medial prefrontal default-mode hypoactivity affecting trait physical anhedonia in schizophrenia. Psychiatry Research: Neuroimaging 171: 155-165.

Reske M, Kellermann T, Habel U, Jon Shah N, Backes V, et al. (2007) Stability of emotional dysfunctions? A long-term fMRI study in first-episode schizophrenia. J Psychiatr Res 41: 918-927.

Rockstroh B, Junghoefer M, Elbert T, Buodo G, Miller GA (2006) Electromagnetic brain activity evoked by affective stimuli in schizophrenia. Psychophysiology [Psychophysiology] 43: 431-439.

Rupp CI, Fleischhacker WW, Kemmler G, Kremser C, Bilder RM, et al. (2005) Olfactory functions and volumetric measures of orbitofrontal and limbic regions in schizophrenia. Schizophr Res 74: 149-161.

Salem JE, Kring AM (1999) Flat affect and social skills in schizophrenia: evidence for their independence. Psychiatry Res 87: 159-167.

Schlenker R, Cohen R, Hopmann G (1995) Affective modulation of the startle reflex in schizophrenic patients. European Archives of Psychiatry and Clinical Neuroscience 245: 309-318.

Schneider F, Habel U, Reske M, Toni I, Falkai P, et al. (2007) Neural substrates of olfactory processing in schizophrenia patients and their healthy relatives. Psychiatry Res Neuroimaging 155: 103-112.

Schneider F, Weiss U, Kessler C, Salloum JB, Posse S, et al. (1998) Differential amygdala activation in schizophrenia during sadness. Schizophr Res 34: 133-142.

Schühoff F, Szöke A, Bellivier F, Turcas C, Villemur M, et al. (2003) Anhedonia in schizophrenia: a distinct familial subtype? Schizophr Res 61: 59-66.

Seok JH, An SK, Lee E, Lee HS, Lee YJ, et al. (2006) Behavioral evidence of blunted and inappropriate affective responses in schizophrenia: Lack of a 'negativity bias'. Psychiatry Res 142: 53-66.

Seubert J, Loughead J, Kellermann T, Boers F, Brensinger CM, et al. (2010) Multisensory integration of emotionally valenced olfactory-visual information in patients with schizophrenia and healthy controls. J Psychiatry Neurosci 35: 185-194.

Simon JJ, Biller A, Walther S, Roesch-Ely D, Stippich C, et al. (2010) Neural correlates of reward processing in schizophrenia - Relationship to apathy and depression. Schizophr Res 118: 154-161.

Schneider F, Gur RC, Gur RE, Shtasel DL (1995) Emotional processing in schizophrenia: Neurobehavioral probes in relation to psychopathology. Schizophr Res 17: 67-75.

Strauss GP, Llerena K, Gold JM (2011) Attentional disengagement from emotional stimuli in schizophrenia. Schizophrenia Research 131: 219-223.

Strauss GPGP, Wilbur RC, Warren KR, August SM, Gold JM (2011) Anticipatory vs. consummatory pleasure: what is the nature of hedonic deficits in schizophrenia? Psychiatry Research 187: 36-41.

Suslow T, Roestel C, Arolt V (2003) Affective priming in schizophrenia with and without affective negative symptoms. European Archives of Psychiatry and Clinical Neuroscience 253: 292-300.

Suslow T, Roestel C, Droste T, Arolt V (2003) Automatic processing of verbal emotion stimuli in schizophrenia. Psychiatry Res 120: 131-144.

Taylor SF, Phan KL, Britton JC, Liberzon I (2005) Neural response to emotional salience in schizophrenia. Neuropsychopharmacology 30: 984-995.

Trémeau F, Antonius D, Cacioppo JT, Ziwich R, Butler P, et al. (2010) Anticipated, on-line and remembered positive experience in schizophrenia. Schizophr Res 122: 199-205.

Trémeau F, Antonius D, Cacioppo JT, Ziwich R, Jalbrzikowski M, et al. (2009) In support of Bleuler: Objective evidence for increased affective ambivalence in schizophrenia based upon evocative testing. Schizophr Res 107: 223-231.

Tso IF, Grove TB, Taylor SF (2010) Emotional experience predicts social adjustment independent of neurocognition and social cognition in schizophrenia. Schizophr Bull 122: 156-163.

Volz M, Hamm AO, Kirsch P, Rey E-R (2003) Temporal course of emotional startle modulation in schizophrenia patients. Int J Psychophysiol 49: 123-137.

Walter H, Kammerer H, Frasch K, Spitzer M, Abler B (2009) Altered reward functions in patients on atypical antipsychotic medication in line with the revised dopamine hypothesis of schizophrenia. Psychopharmacology (Berl) 206: 121-132.

Waltz JA, Schweitzer JB, Gold JM, Kurup PK, Ross TJ, et al. (2009) Patients with schizophrenia have a reduced neural response to both unpredictable and predictable primary reinforcers. Neuropsychopharmacology 34: 1567-1577.

Waltz JA, Schweitzer JB, Ross TJ, Kurup PK, Salmeron BJ, et al. (2010) Abnormal responses to monetary outcomes in cortex, but not in the basal ganglia, in schizophrenia. Neuropsychopharmacology 35: 2427-2439.

Wynn JK, Horan WP, Kring AM, Simons RF, Green MF (2010) Impaired anticipatory event-related potentials in schizophrenia. Int J Psychophysiol 77: 141-149.

Yee CM, Mathis KI, Sun JC, Sholty GL, Lang PJ, et al. (2010) Integrity of emotional and motivational states during the prodromal, first-episode, and chronic phases of schizophrenia. J Abnorm Psychol 119: 71-82.
